# Phase 2 trial of ixazomib in patients with relapsed multiple myeloma not refractory to bortezomib

**DOI:** 10.1038/bcj.2015.60

**Published:** 2015-08-14

**Authors:** S K Kumar, B LaPlant, V Roy, C B Reeder, M Q Lacy, M A Gertz, K Laumann, M A Thompson, T E Witzig, F K Buadi, C E Rivera, J R Mikhael, P L Bergsagel, P Kapoor, L Hwa, R Fonseca, A K Stewart, A Chanan-Khan, S V Rajkumar, A Dispenzieri

**Affiliations:** 1 Division of Hematology, Mayo Clinic, Rochester, MN, USA; 2 Department of Biostatistics, Mayo Clinic, Rochester, MN, USA; 3 Division of Hematology and Oncology, Mayo Clinic, Jacksonville, FL, USA; 4 Division of Hematology and Oncology, Mayo Clinic, Scottsdale, AZ, USA

## Abstract

This phase 2 trial was designed to evaluate ixazomib, an orally bioavailable proteasome inhibitor, in patients with myeloma who have limited prior exposure to bortezomib. Thirty-three patients with relapsed multiple myeloma were enrolled. Ixazomib was given at 5.5 mg weekly for 3 of 4 weeks. Dexamethasone was added for lack of a minor response (MR) by end of cycle 2 or lack of a partial response (PR) by end of cycle 4 or for disease progression at any time. Median age was 69 years; patients had a median of two prior therapies (range 1–7). A grade 3 or 4 adverse event considered at least possibly related to drug was seen in 19 (59%) and 6 (19%) patients, respectively. The most common adverse events were thrombocytopenia, fatigue, nausea and diarrhea. Dexamethasone was initiated in 22 (67%) patients, 17 for not reaching the desired response and 5 for progression. Response (⩾PR) to single agent was seen in five patients within four cycles of therapy including three patients with PR, one patient with complete response (CR) and one patient with stringent CR. Six additional patients with either an MR (2) or SD (4) achieved a PR after addition of dexamethasone, translating to an overall response rate of 34%.

## Introduction

Proteasome inhibition has become an important therapeutic strategy in multiple myeloma (MM), for newly diagnosed as well as relapsed disease, and particularly in patients with certain cytogenetic abnormalities associated with aggressive disease behavior.^1, 2^ Bortezomib was the first proteasome inhibitor to be approved for the treatment of cancer, and has changed the treatment paradigm in MM.^3, 4, 5, 6^ More recently, another proteasome inhibitor, namely carfilzomib, was approved for treatment of relapsed myeloma based on promising results seen in a large phase 2 study.^7, 8^ Proteasome inhibitors when combined with immunomodulatory drugs, such as lenalidomide or alkylating agents, have resulted in some of the most effective treatment regimens in myeloma to date.^9, 10, 11^ Two major stumbling blocks to widespread use of this class of drugs have been the risk of peripheral neuropathy associated with bortezomib administration and the need for parenteral administration.^12^ The risk of peripheral neuropathy with bortezomib has been mitigated to some extent with the weekly schedule and the use of subcutaneous administration with this drug.^13, 14^ Moreover, results of the studies so far suggest a very low rate of neuropathy among patients receiving the newer proteasome inhibitor carfilzomib. However, the need for a clinic visit for subcutaneous bortezomib or intravenous carfilzomib adds to the disease-related burden for patients, especially those on long-term therapy.

Ixazomib citrate (MLN9708) is an investigational inhibitor of the 20S proteasome that represents the first orally bioavailable proteasome inhibitor to be evaluated for the treatment of MM.^15^ Ixazomib citrate is a modified peptide boronic acid and is the citrate ester of ixazomib (MLN2238), the biologically active moiety. Ixazomib citrate rapidly hydrolyzes to ixazomib upon contact with aqueous solution or plasma. Ixazomib preferentially binds the β_5_ site of the 20 S proteasome at lower doses, with inhibition of the β_1_ and β_2_ sites at higher concentrations. Compared with bortezomib, nonclinical studies have shown that ixazomib has a faster dissociation rate from the proteasome. Ixazomib has demonstrated antitumor activity in a range of tumor xenograft models, including MM models.^16, 17^ Preclinical studies have shown activity in myeloma cells resistant to bortezomib as well as synergistic anti-myeloma activity when combined with dexamethasone and lenalidomide. In clinical trials, ixazomib has shown promising activity as a single agent in patients with relapsed and refractory MM, with very low rates of peripheral neuropathy observed in the single-agent trials.^18, 19, 20^ Given that the majority of patients in the early trials had been exposed previously to bortezomib, we designed this trial to better understand the efficacy of single agent ixazomib in patients with relapsed MM with limited exposure to bortezomib and also to examine the utility of adding dexamethasone to ixazomib.

## Patients and methods

### Study design

This open-label phase 2 study evaluated the safety, tolerability and efficacy of weekly oral ixazomib citrate in patients with relapsed MM who either had no exposure to proteasome inhibitors or had limited (no more than six cycles) exposure to a bortezomib-containing regimen. It also explored the utility of adding weekly dexamethasone to ixazomib in patients with suboptimal response to single agent ixazomib. The study enrolled patients from January 2012 to October 2012. The study was performed in accordance with the provisions of the Declaration of Helsinki, the International Conference on Harmonization, and the Guidelines for Good Clinical Practice, and with approval of the Mayo Clinic Institutional Review Board. The study was registered at www.clinicaltrials.gov as NCT01415882.

### Study objectives

The primary objective of the study was to determine the confirmed overall response rate (ORR) (⩾PR (partial response)) of ixazomib, used as a single agent in patients with relapsed MM, who were proteasome inhibitor naïve or had received less than six cycles of therapy with bortezomib, and were not refractory to bortezomib. The secondary objectives included assessment of ORR of ixazomib in combination with dexamethasone, when dexamethasone was added for lack of response or for disease progression, and measurement of event-free survival and overall survival following treatment with ixazomib with dexamethasone added for lack of response or progression.

### Patient selection

The study enrolled patients, 18 years of age or older, with MM that had relapsed after at least one previous therapy. Patients were required to have measurable disease (serum M-protein ⩾1 g/dl or urine M-protein ⩾200 mg/24 h or involved free light chain level ⩾10 mg/dl provided the serum free light chain ratio was abnormal), Eastern Cooperative Oncology Group performance status of 0–2, adequate hematologic (absolute neutrophil count ⩾1000/mm^3^, platelets ⩾75 000/mm^3^), hepatic (total bilirubin ⩽1.5 × upper limit of normal, alanine/aspartate aminotransferase ⩽3 × upper limit of normal) and renal (creatinine clearance ⩾30 ml/min) function. Patients with grade ⩾3 peripheral neuropathy or grade 2 with pain, grade >1 diarrhea, or who had major surgery or serious infection within 14 days prior to start of therapy were excluded. Patients receiving systemic treatment with strong CYP1A2 inhibitors or strong inhibitors/inducers of CYP3A within 14 days were excluded. Other factors that precluded participation in the trial included uncontrolled cardiovascular conditions (including uncontrolled hypertension, uncontrolled cardiac arrhythmias, symptomatic congestive heart failure, unstable angina, or myocardial infarction within the past 6 months), known human immunodeficiency virus positivity, known hepatitis B surface antigen-positive status, or known or suspected active hepatitis C infection, and known allergy to any of the study medications, their analogues, or excipients in the various formulations. Other comorbidities or severe pre-existing illness that in the treating physician's opinion could interfere with oral absorption and/or tolerance of ixazomib citrate excluded patients from participation.

### Drug administration

Ixazomib was administered orally at a dose of 5.5 mg on days 1, 8 and 15 of a 28-day cycle. Dexamethasone at a dose of 20 mg orally was added on days 1, 2, 8, 9, 15 and 16 of the 28-day cycle for lack of a minor response (MR) by end of cycle 2, lack of a PR by end of cycle 4 or if there was disease progression at any time. Dose modifications were made for ixazomib-related toxicities with successive reductions in its dose to 4, 3, 2 mg followed by discontinuation if the 2 mg dose was not tolerated. Patients who had confirmed progression despite addition of dexamethasone were taken off study.

Prophylactic anti-emetics were not initially planned, but given the incidence of nausea among the initial 12 patients, the study was amended to allow prophylactic 5 HT3 antagonists prior to each dose of ixazomib. Prophylactic antidiarrheals were not used; however, the administration of antidiarrheals was allowed after infectious causes were excluded. Topical steroids and other symptomatic measures were permitted for management of any skin rash.

### Assessments

Adverse events (AEs) were graded using the National Cancer Institute's Common Terminology Criteria for AEs, version 4.0. Myeloma disease response was done in accordance with the International Myeloma Working Group uniform criteria, incorporating the additional category of MR. All response categories required confirmation of the required tests with the exception of the bone marrow used for complete response (CR) determination. At any point in treatment, patients suspected of progressive disease had response assessments repeated to confirm disease progression, done at least 1 week apart.

### Statistical analyses

The primary endpoint of this study was the ORR with single agent ixazomib, where a success was defined as sCR, CR, VGPR or PR noted as the objective status on two consecutive evaluations while receiving single agent ixazomib. All patients meeting the eligibility criteria, who signed a consent form and received at least one dose of the drug were evaluable for response, with the exception of patients who are determined to be a major treatment violation. The sample size for the study was calculated using one-stage binomial design. With 29 evaluable patients, the study provided 91% power to test the null hypothesis that the ORR is at most 10% versus the alternative hypothesis that the ORR is at least 30%, with a one-sided significance level of α=0.06. An additional four patients were enrolled to account for ineligible patients and protocol violations, for a total of 33 patients. For toxicity assessment, all patients who received at least one dose of study drug were included in the analysis. Overall survival was defined as the time from study entry to death due to any cause. Event-free survival was defined as the time from study entry to disease progression while receiving ixazomib and dexamethasone, death due to any cause or subsequent treatment for myeloma. Patients who went off study but never received dexamethasone were censored at the off-study date.

## Results

### Patients

**Figure 1:**
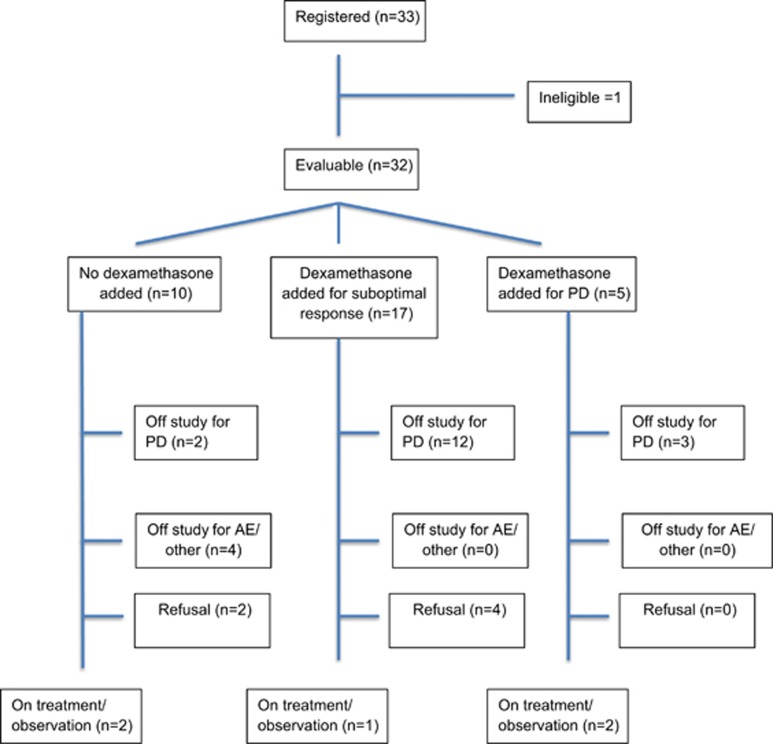
Patient disposition across the entire study including addition of dexamethasone.

Thirty-three patients were enrolled, and one patient was considered ineligible and excluded from all analysis. The median age was 69 years and 53% were male. The median duration from diagnosis was 57 months (range 14 months to 12.3 years) and patients had a median of two prior therapies (range 1–7). The baseline characteristics at study entry are described in Table 1. Prior therapies included IMiDs (88%), bortezomib (28%) and stem cell transplant (59%). At the time of data cutoff, 19 (59%) patients had progressed and 27 (84%) were alive, with a median follow-up of 22 months (range 1–29). Five remain on therapy; reasons for drug discontinuation were disease progression (17), refusal (6), AE (3) and physician discretion (1). Patient disposition is outlined in Figure 1.

### Response to therapy and survival

**Figure 2:**
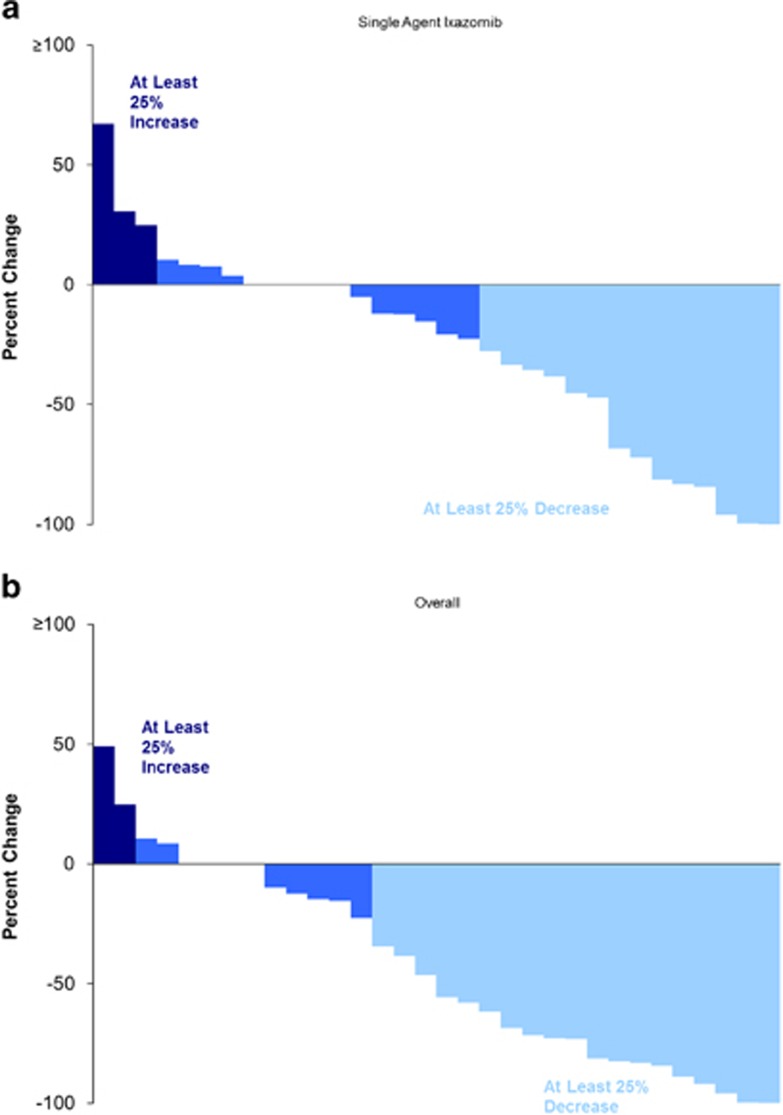
(**a**) Waterfall plot of the distribution of depth of the response observed for single agent ixazomib and (**b**) waterfall plot of the distribution of depth of the response observed across the entire study.

Overall, 11 (34%) patients achieved a PR or better across the entire trial, with or without the addition of dexamethasone, including one patient each with CR and sCR. An additional two patients achieved a MR. Dexamethasone was initiated in 22 (67%) patients, 17 for not reaching the desired response (MR by end of two cycles and PR by end of four cycles) and in five patients dexamethasone was added for progression. The timing of dexamethasone and the responses before and after addition of dexamethasone is detailed in Table 2. The response rates were similar among patients irrespective of the prior exposure to bortezomib. The response rates grouped by refractoriness to lenalidomide, bortezomib sensitivity, as well as FISH-based risk status are as shown in Table 3. No impact of age on the response rate was observed in the current study. A waterfall plot highlighting the depth of the responses observed is shown in Figures 2a and b, respectively for single agent ixazomib and for the overall study.

**Figure 3:**
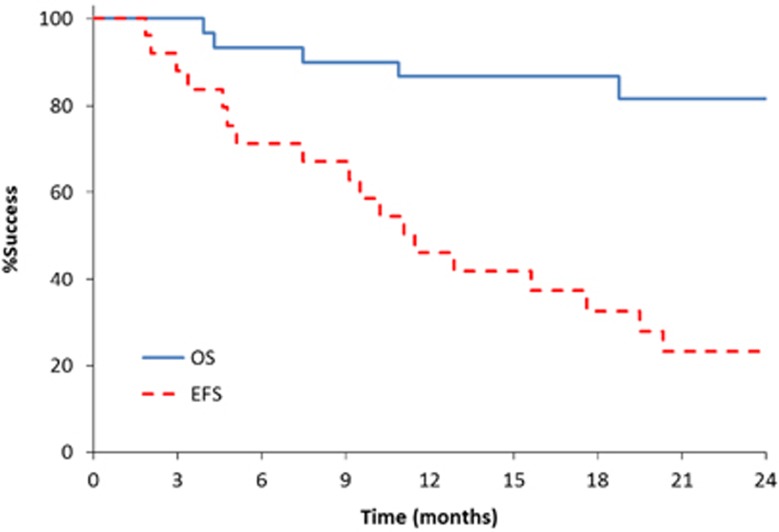
Overall survival (OS) and event-free survival (EFS) for the entire study population.

The median event-free survival was 11.5 months (95% confidence interval: 5.1–19.5) and 6 month overall survival was 93% (Figure 3). The event-free survival was not significantly different for patients previously exposed to bortezomib, compared with the bortezomib naïve patients. The median duration of response among the 15 patients with a MR or better was 17.4 months (95% confidence interval: 7.4-NR).

### Dose intensity and AEs

Patients received a median of eight cycles of therapy (range, 1–30) across the trial; 19 and 16 patients received at least four and eight cycles, respectively and 12 patients stayed on trial for more than 12 cycles. Overall, 336 cycles of treatment were delivered, with a dose reduction required for 21 (6%) cycles. A median of 100% (range, 33–100) of the intended dose of ixazomib was delivered, whereas a median of 100% (range, 33–133) of intended dose of dexamethasone was delivered among the 22 patients started on dexamethasone. The median dose (per cycle) of ixazomib at the time of discontinuation among the 27 patients who have gone off therapy was 12 mg (range, 5.5–16.5).

**Figure 4:**
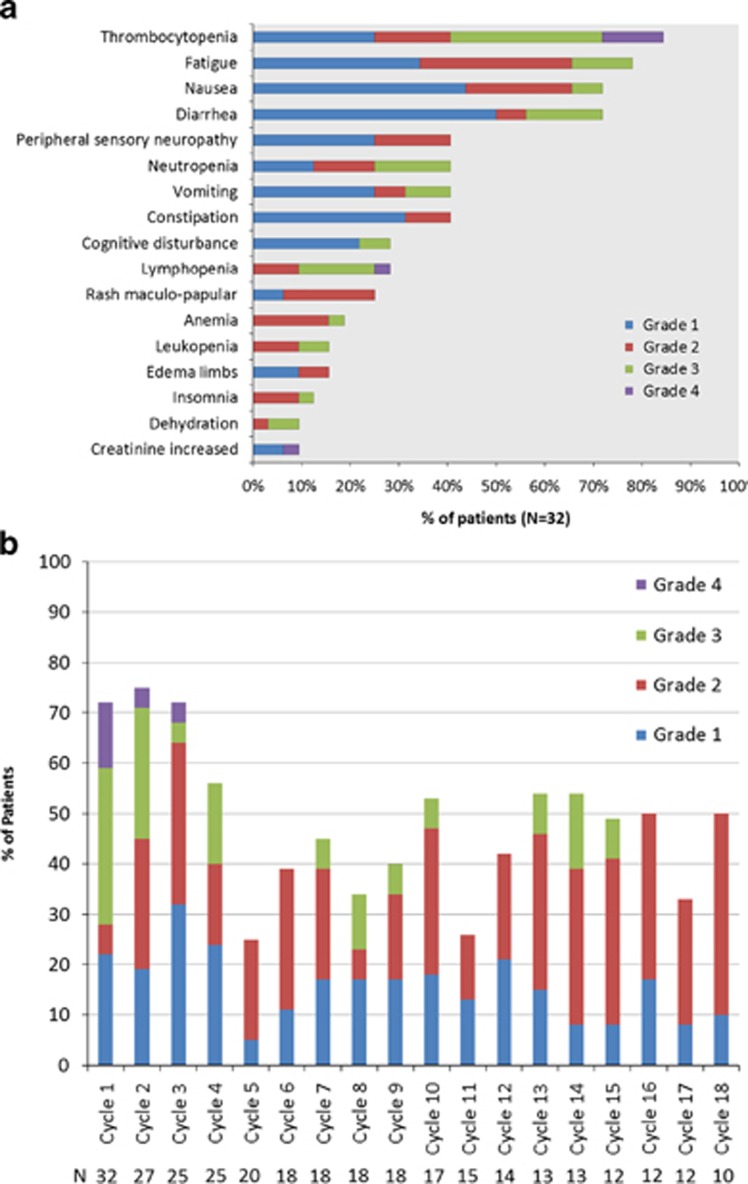
(**a**) The distribution of all grades of toxicities considered at least possibly related to the drug administration. (**b**) The incidence of hematological toxicity across individual cycles, highlighting lack of any cumulative hematological toxicity.

An AE of any grade, that was considered at least possibly related, was reported in 100% of the patients. A grade 3 or 4 AE considered at least possibly related to drug was seen in 19 (59%) and 6 (19%) patients, respectively; there were no deaths on study. The most common AEs observed included thrombocytopenia, fatigue, nausea and diarrhea. Peripheral neuropathy possibly related to the drug was seen in eight patients (grade 1) and five patients (grade 2), respectively. Figure 4a provides the distribution of all grades of toxicities considered at least possibly related to the drug administration. No cumulative hematological toxicity was observed across the entire trial (Figure 4b). Three patients went off study due to an AE; these included elevated serum creatinine, thrombotic thrombocytopenic purpura and physician discretion.

Overall, 22 patients had dexamethasone added per protocol and 173 of the 336 cycles administered contained dexamethasone. We compared the toxicity profile between the cycles containing dexamethasone and those with ixazomib as a single agent. Less hematologic and gastrointestinal (GI) toxicity was seen in patients receiving the combination than in those receiving ixazomib alone (Table 4).

## Discussion

Proteasome inhibitors have become an integral part of myeloma therapies in the upfront setting as well as in the relapsed setting. Increasingly, this class of drugs is being utilized in combination with other myeloma drugs, both new and old. Introduction of an oral proteome inhibitor can have a significant impact on the management of myeloma, as this would allow for all-oral combination regimens incorporating proteasome inhibitors and immunomodulatory drugs (IMiDs). Ixazomib has been studied as a single agent, given once or twice weekly, in the initial phase 1 studies.^18, 20^ Subsequent studies have explored the combination of ixazomib with lenalidomide in newly diagnosed myeloma, where the combination had high efficacy as well as adequate tolerability to allow for long-term therapy as a maintenance agent.^19^ The current study was designed to ask two important questions: what is the single-agent activity of ixazomib among patients with limited prior exposure to proteasome inhibitors and the value of combining ixazomib with dexamethasone. The latter is particularly relevant, as most of the currently used combinations include corticosteroids, and such a two-drug regimen can have value in situations that calls for less intense and more convenient therapies as in older or frailer patient.

The current study confirms the single-agent activity of ixazomib that has been observed in the initial phase 1 trial, where 24% of the patients treated at the 5.5 mg weekly dose of ixazomib had a PR or better.^18^ It is further highlighted by the deep responses of sCR and CR seen in one patient each. It is difficult to directly compare the proportion of responses between the two studies, given that patients in the current study had dexamethasone added for lack of adequate responses by two or four cycles of therapy or progression at any time. The response rates also need interpretation in the context of initial trials of bortezomib, where it was used as a single agent in patients with relapsed MM. In the phase 3 APEX trial, patients receiving bortezomib on days 1, 4, 8 and 11 of a 3-week cycle had overall best response rate of 38%, representing the best response across the trial in a group of patients with median of two prior therapies, which primarily included alkylators, anthracyclines, high dose therapy or thalidomide.^6^ Majority of the patients in the current study in contrast had been refractory to lenalidomide and had prior high dose therapy. The approach of adding dexamethasone for suboptimal response was also evaluated in the SUMMIT trial, where patients received bortezomib on days 1, 4, 8 and 11 of a 3-week cycle with dexamethasone for progressive disease after two cycles or stable disease after four cycles.^21^ The ORR was 27% with monotherapy and 18% of the 78 patients who had dexamethasone added had a MR or better. In the current study, we had more stringent criteria for addition of dexamethasone leading to earlier addition, and among the 22 patients, who received dexamethasone eventually, an additional four patients had a PR and another four had an MR. The ORR among the entire cohort with this on-demand approach of adding dexamethasone was 34% with a clinical benefit rate of 48% including the four patients with an MR. Responses did not differ significantly depending on prior exposure to bortezomib, but the overall numbers are low and it is important to note that both patients achieving a CR had no prior bortezomib exposure.

The main toxicities that we observed here are in line with the previous experience with this drug, and included nausea, thrombocytopenia and fatigue.^18, 20^ We noticed more nausea in the beginning of the study and instituted prophylactic anti-emetic prior to each dose, which abrogated this problem in the majority of the patients. Interestingly, the overall GI toxicity was less in the cycles that also included dexamethasone, which may reflect the anti-emetic effect of the drug or the fact that dexamethasone was added in later by which time the GI toxicity may have been managed more efficiently. The thrombocytopenia was typically noted mid-cycle and recovered prior to initiation of a new cycle in the vast majority of patients, and no cumulative effect was seen across the study. Overall, the frequency of dose reduction was low at 6% across the 336 cycles over the study. Rate of peripheral neuropathy was low and predominantly grade 1 or 2; eight patients with grade 1 and 5 patients with grade 2.

In conclusion, ixazomib has promising single-agent activity in relapsed myeloma along with a favorable toxicity profile. Addition of dexamethasone significantly enhances the response rates demonstrating an important role for this two-drug combination. Given the convenience of oral route and once weekly dosing, this regimen can have a role in the management of the older patients and the more frail patient as well as in patients with more indolent relapses. Given the results with delayed addition of dexamethasone, it is likely that initiation of therapy along with dexamethasone can lead to higher response rates as well as deeper responses. Another cohort of patients is being enrolled currently, to examine the utility of adding dexamethasone from the beginning.

## Figures and Tables

**Table 1:** Baseline characteristics

	N=*32*
Median age, years (range)	69 (52–82)
Age ⩾75 years, *n* (%)	8 (25%)
Male, *n* (%)	17 (53%)

*ISS disease stage at registration, n (%)*	
I	11 (34%)
II	14 (44%)
III	7 (22%)

*MM subtype, n (%)*	
IgG	23 (72%)
IgA	6 (19%)
IgD	2 (6%)
Light chain	1 (3%)
Median creatinine clearance, ml/min (range)	1 (0.5–1.9)
Abnormal metaphase cytogenetics, *n* (%)	10 (31%)

*FISH abnormalities, n (%)*	
del 13	2 (6%)
del 17p	4 (13%)
*t*(4;4)	0
*t*(14;6)	0
*t*(11;14)	9 (28%)
Median number of therapies (range)	2 (1–7)
Bortezomib exposed, *n* (%)	9 (28%)
Lenalidomide refractory, *n* (%)	25 (78%)
Prior stem cell transplant, *n* (%)	19 (60%)

Abbreviations: FISH, fluorescence *in situ* hybridization; Ig, immunoglobulin.

**Table 2:**
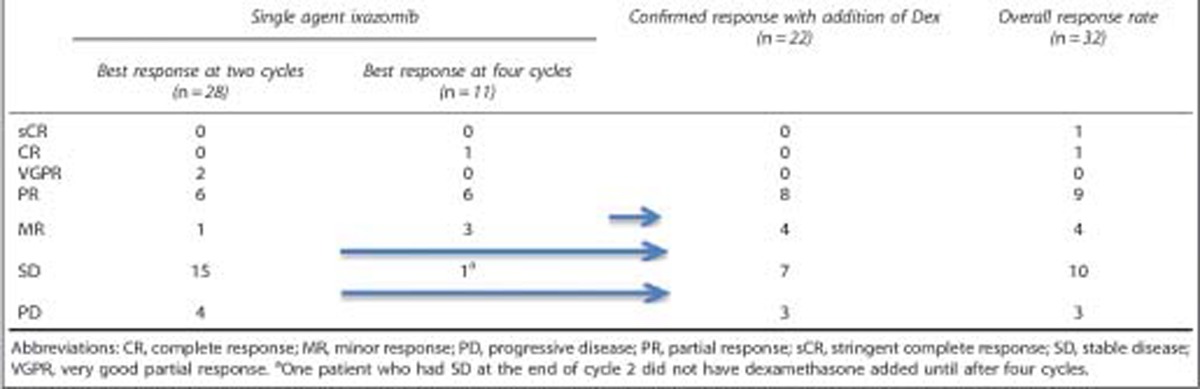
Response to treatment with single agent ixazomib and effect of addition of dexamethasone

**Table 3:** Responses by subgroups

	*Age*		*mSMART risk*		*Prior bortezomib*		*Prior lenalidomide*	
	*<65 (*n=*13)*	⩾*65 (*n=*19)*	*High (*n=*4)*	*Standard (*n=*28)*	*Yes (*n=*9)*	*No (*n=*23)*	*Yes (*n=*25)*	*No (*n=*7)*
*Response to single agent ixazomib*								
sCR	0	1	0	1	0	1	1	0
CR	0	1	0	1	0	1	1	0
VGPR	0	0	0	0	0	0	0	0
PR	1	2	1	2	1	2	1	2
MR	3	1	0	3	0	3	3	0

*Overall response*								
sCR	0	1	0	1	0	1	1	0
CR	0	1	0	1	0	1	1	0
VGPR	0	0	0	0	0	0	0	0
PR	4	5	2	7	3	6	6	3
MR	1	3	0	3	2	2	4	0

Abbreviations: CR, complete response; MR, minor response; PR, partial response; sCR, stringent complete response; VGPR, very good partial response.

**Table 4:** Impact of dexamethasone addition on toxicity profile

	*Ixazomib alone (*N=*163 cycles)*	*Ixazomib+dexamethasone (*N=*173 cycles)*	P*-value (Fisher's exact)*
Grade 3+ heme	30 (18%)	7 (4%)	<0.001
Grade 3+ non-heme	15 (9%)	7 (4%)	0.08
Grade 3+ GI	7 (4%)	1 (0.6%)	0.03
All grade GI	124 (76%)	84 (49%)	<0.001

Abbreviation: GI, gastrointestinal.

## Notes

SKK obtained research support for clinical trials from Celgene, Millennium, Novartis, Janssen, Sanofi. JRM obtained research funding from Onyx Celgene Abbvie and Sanofi. PLB worked as consultant for Amgen, Mundipharma, Sanofi-Aventis, Janssen. He obtained research support from Novartis, Constellation Pharmaceuticals. PK obtained research support for clinical trials from Onyx. RF received consulting fees from Celgene, Genzyme, BMS, Bayer, Lilly, Onyx, Binding Site, Novartis, Sanofi, Millennium and AMGEN. He also has sponsored research from Onyx. He is also a member of the Scientific Advisory Board of Applied Biosciences. AKS worked as consultant for Celgene, Janssen, Novartis. AD obtained research support for clinical trials from Pfizer, Jannsen, Millenium, Alnylam, and Celgene. The remaining authors declare no conflict of interest.
